# Insights into hydrophobic waste valorization for the production of value‐added oleochemicals

**DOI:** 10.1111/1751-7915.14122

**Published:** 2022-08-05

**Authors:** Sachin Vyas, Leonidas Matsakas, Ulrika Rova, Paul Christakopoulos, Alok Patel

**Affiliations:** ^1^ Biochemical Process Engineering Division of Chemical Engineering Department of Civil Environmental, and Natural Resources Engineering Luleå University of Technology Luleå Sweden

Increasing demands and changes in the living style due to overpopulation has increased the amount of waste generated globally. The waste management system must meet the criteria of the United Nations' sustainable development goals, including being cost‐effective, societally useful and environmentally safe (United Nations, 2015). Such systems are largely dependent on the nature, source, composition and usability of the waste (Orjuela & Clark, 2020). A global major goal is to valorize waste streams while concurrently producing bio‐products towards a circular economy and long‐term sustainability. Biorefineries are sustainable systems that combine biomass conversion processes with the production of bioenergy (power, biofuels and chemicals) and bio‐products (food, feed and materials) (Ubando et al., 2020; Vyas, Patel, et al., 2022b). Hydrophobic wastes of lipid nature originate either from household and hospitality sectors (hotels, restaurants, canteen and catering) or from various industries for vegetable oil, fish, animal meat and food processing (Patel, Delgado Vellosillo, et al., 2022b). These wastes often contain a variety of free fatty acids or esters with varying carbon chain lengths (C4–C24) (Fickers et al., 2005), and they are useful feedstocks for microorganisms since they are inexpensive and abundant. Over the last few decades, the production of biodiesel has dominated the processes to valorize hydrophobic waste streams, there is, however, a challenge to develop additional processes that target high‐value goods production to ensure a financially viable biorefinery. The establishment of such a process will provide the space for new value‐added products in the market. The present opinion article outlines the current knowledge on various oleaginous microorganisms for the valorization of hydrophobic waste. The different challenges and approaches to use biotechnological interventions for establishing a sustainable cycle for the simultaneous treatment of hydrophobic waste and production of value‐added compounds are presented.

## CURRENT MANAGEMENT OF HYDROPHOBIC WASTES AND THEIR UTILIZATION TOWARDS A CIRCULAR ECONOMY

The amount of waste coming from the household and hospitality sectors is affected by dietary preferences, customs, traditions and population behaviour from region to region (Yu et al., 2018). The current global market of vegetable oils for food sectors is anticipated to increase by 1.5 times by the year 2024 as compared to the year 2015 due to rising needs brought on by a growing global population (Ahmad et al., 2020). Due to the lack of strict following of the guidelines or available systems to appropriately utilize such waste, majority of them are directly released into the nature (Patel, Delgado Vellosillo, et al., 2022b). This has a significant adverse impact on climate as lipids form a thin layer over natural water bodies, depleting dissolved oxygen and harming aquatic life (Patel, Delgado Vellosillo, et al., 2022b). It also affects the terrestrial environments via leaching through the soils and contaminating the groundwater resources (Hosseinzadeh‐Bandbafha et al., 2022). Most developing countries lack a system for collecting and managing such wastes.

Waste cooking oil is largely used in biodiesel production or other industrial purposes such as the production of hydrogenated vegetable oil, soaps and animal feed, and has been extensively reviewed previously (Orjuela & Clark, 2020). Food safety control system guidelines in many countries include measures to restrict cooking oil reuse, and that must be discarded after a certain number of cycles based on its quality (Loizides et al., 2019). It was also banned from being used as animal feed by the EU in 2002 due to carcinogenic and other harmful substances bioaccumulating in humans (Zhao et al., 2021). Wastewaters released from various industries such as oil refineries, lubricant manufacturing and food processing industries during different stages of oil processing contain several pollutants with high organic loading (COD), different salts and surfactants. Such wastewater is treated in two major routes. The primary treatment includes physical methods such as coagulation, filtration and flocculation for solid particle removal. Most of the lipids and grease in wastewater are not retained properly in the primary treatment. The secondary treatment involves biological methods using aerobic or anaerobic digestion. The high lipid and salt contents in such waste limit the application of anaerobic digestion in the treatment due to clogging (Mannacharaju et al., 2020).

Oleaginous microorganisms belonging to yeasts, microalgae, fungi, protists and bacteria are known for their high lipid production (more than 20% w/w of cell dry weight) (Patel et al., 2020; Vyas & Chhabra, 2017). Oleaginous yeasts such as *Trichosporon fermentans*, *Cryptococcus curvatus*, *Wickerhamomyces anomalus* and *Yarrowia lipolytica* have been utilized for lipid accumulation using wastes containing hydrophobic substrates (Arous et al., 2017; Patel & Matsakas, 2019; Yu et al., 2018). Most of these studies were limited to the production of microbial lipids as feedstock for biodiesel without studying the co‐production of other bio‐based products. The biodiesel industry is still in a nascent stage as it cannot compete with the current crude oil prices and significant research efforts are still needed to make it an economically viable product (Sreeharsha & Mohan, 2020). Apart from biodiesel, certain microbial lipids with a higher percentage of saturated fatty acids can also be used in the food industry as a substitute for cocoa butter (Wei et al., 2017). Microbial lipids have also been demonstrated for their importance in biopolymer (polyester, polyurethane, polyether and polyolefin), and fatty acid alcohol production (Vasconcelos et al., 2019). However, as described above, most hydrophobic wastes derived from industries have also high levels of salinity, which limits the use of yeasts or freshwater microalgae in the process.

**FIGURE 1:**
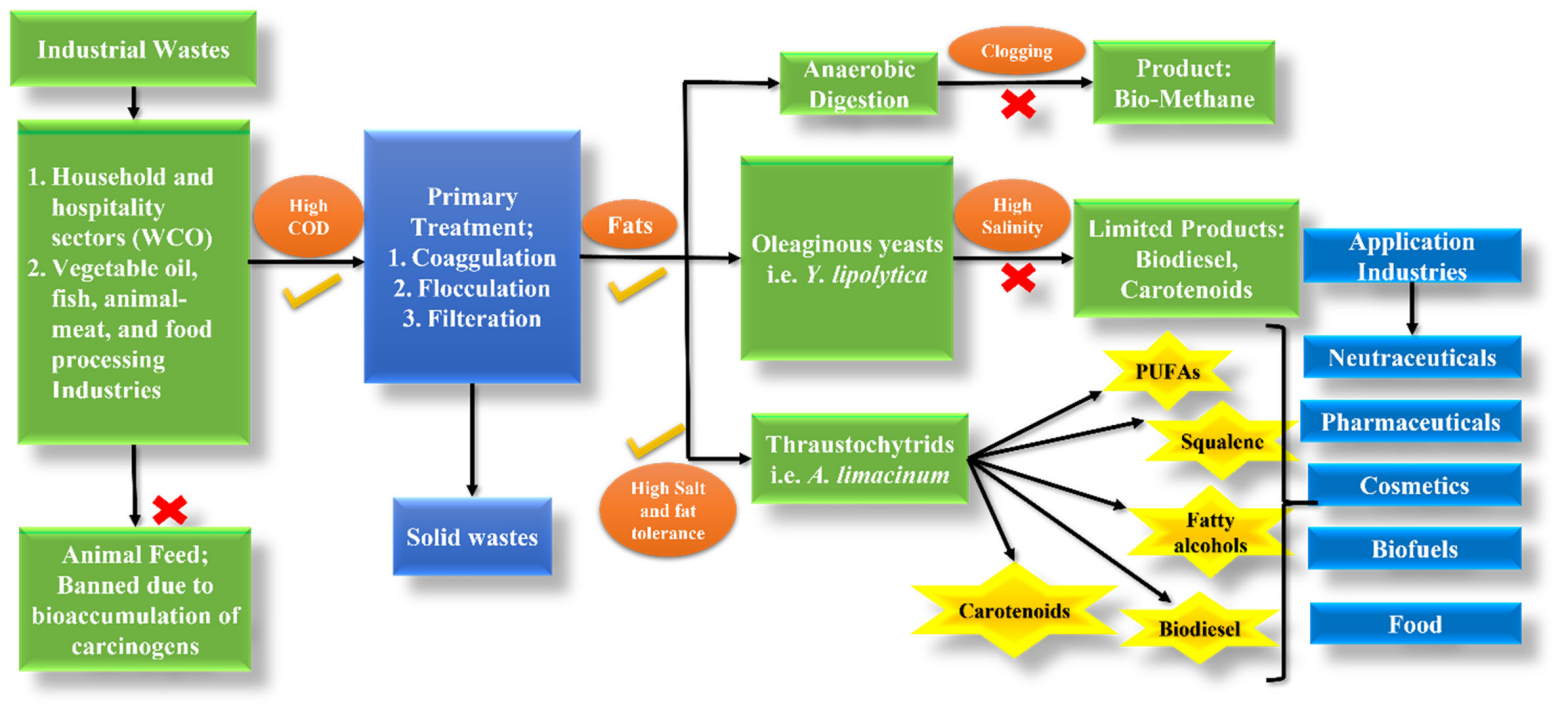
Insights into hydrophobic waste valorization by oleaginous microorganisms.

In recent years, marine and salt‐tolerant microorganisms such as thraustochytrids have gained significant attention as promising multi‐product single‐cell biorefinery due to their ability to accumulate a high lipid content, wide substrate range, salt‐tolerance and production of oleochemicals  [carotenoids, squalene, long‐chain polyunsaturated fatty acids (PUFAs) such as docosahexaenoic acid (DHA), eicosapentaenoic acid (EPA) and docosapentaenoic acid (DPA)] (Aasen et al., 2016; Patel, Bettiga, et al., 2022a). PUFAs cannot be synthesized by humans naturally, making them one of the essential fatty acids which in turn resulted in increased market value. PUFAs have been recognized as an effective treatment for various human diseases related to respiratory, cardiac and immune systems (Jia et al., 2021). Squalene, a triterpene synthesized in higher amounts by thraustochytrids such as *Aurantiochytrium limacinum* species is important for pharmaceuticals and cosmetics industries (Vyas, Bettiga, et al., 2022a). In general, thraustochytrids have been effective sources of such molecules, being one of the most important alternatives to fish oils that suit vegetarian and vegan diets and are suitable for meeting the high demands for such molecules (Patel et al., 2020) (Figure 1).

Despite all the technological advancements, the production cost of such oleochemicals remain high to compete in the market with existing conventional sources (Sreeharsha & Mohan, 2020). Efficient development of downstream processing is also an important aspect of commercializing such compounds. Estimation of overall production cost through total life cycle or techno‐economic analysis is an important tool to understand how the different stages of the process affect the process viability. Apart from process economics, process sustainability is also important to be addressed. Life cycle assessment (LCA) is a commonly used methodology for calculating the impact of energy consumption, emissions and natural resource use at all stages of a product's life cycle (Jain et al., 2022). To justify sustainability, the optimum situation with the highest profit at the lowest financial and environmental cost must be identified. Cost‐cutting can also be achieved through a recovery of resources at every step of the process. To date, only laboratory‐scale reports have been produced for such waste streams and the reports on LCA or TEA analysis for the valorization of industrial wastes containing hydrophobic substrates are scarce. More studies on the understanding of the process and production of marketable chemicals at large scales are needed. Such studies will pave the way for reduced GHG emissions, reduced production price, employment generation through several application sectors, and smaller carbon footprints (Venkata Mohan et al., 2016).

## EX‐NOVO LIPID SYNTHESIS PATHWAY AND OLEAGINOUS MICROORGANISMS

In oleaginous microorganisms, there are primarily two types of lipid production pathways: *de‐novo* and *ex‐novo*. The *de‐novo* lipid synthesis pathway (also known as the Kennedy pathway) has been extensively investigated and usually accumulates lipids using a hydrophilic substrate such as glucose, while the external medium is depleted of macro‐elements (mainly nitrogen and phosphorous) (Papanikolaou & Aggelis, 2011). In the case of *ex‐novo* synthesis, hydrophobic substrates are first converted to free fatty acids through various microbial lipolytic mechanisms, followed by active transportation inside the microbial cells (Papanikolaou & Aggelis, 2011). The availability of only hydrophobic substrates to the microorganism in an external medium makes the process growth‐oriented and does not require nitrogen limitations (Tzirita et al., 2020).

Only a few yeasts belonging to the genera *Yarrowia*, *Trichosporon*, *Candida*, etc., and thraustochytrids species belonging to *Aurantiochytrium* genus (previously known as *Schizochytrium*) can grow and utilize lipid‐containing medium as a sole carbon source (Papanikolaou & Aggelis, 2010; Patel, Delgado Vellosillo, et al., 2022b). Extracellular lipases (E.C. 3.1.1.3) play an important role in the utilization of triacylglycerols (TAGs) available in the medium. Lipases of the oleaginous yeast *Y. lipolytica* are well identified, characterized, and the *ex‐novo* lipid synthesis pathway is thoroughly investigated (Fickers et al., 2005; Thevenieau et al., 2018). Recent studies also highlight increasing interest in thraustochytrid species for their capability to grow on hydrophobic substrates by expressing intra‐ and extracellular lipases and accumulation of industrially important oleochemicals (Ishibashi et al., 2019; Laddha et al., 2021; Patel, Delgado Vellosillo, et al., 2022b). However, the exact mechanism, enzymes involved, and pathways for utilization of hydrophobic in thraustochytrids yet remain unknown.

In general, heterotrophic oleaginous microorganisms that are capable of growing on a hydrophobic substrate and producing lipases will consume the substrate in three major steps; (1) emulsification (contact between the substrate and cells via surfactant or liposan emulsifier production) or adhesion of cells to hydrophobic substrate droplets, (2) Hydrolysis by expression of lipolytic enzymes and (3) Uptake of the free fatty acids using active transport inside the cells (Fickers et al., 2005; Thevenieau et al., 2018; Tzirita et al., 2020). The length of the carbon chain as well as the degree of unsaturation influence fatty acid transport into cells (Thevenieau et al., 2018). Among the oleaginous microorganisms described, there is still a controversy about how these fatty acids are taken up by the cells (Thevenieau et al., 2018). The transported fatty acids are processed through the *β‐*oxidation pathway inside peroxisomes, transforming these fatty acids into the desired lipid profile, generally synthesized by the cells. Acyl‐CoA oxidases (AOX) play a major role in the metabolism of fatty acids and usually represent a rate‐limiting step with a different range of substrate specificity (Thevenieau et al., 2018). Further, fatty acids are metabolized via a multi‐step metabolic pathway process. Each mole of acetyl‐CoA generation contributes one mole of each of FADH_2_ and NADH. The acetyl and acyl‐CoA thus synthesized are utilized either in the cell growth as central metabolic precursors or as building blocks for the oleochemicals (i.e. TAGs) (Papanikolaou & Aggelis, 2010). In some species such as *C. lipolytica* the lipids are accumulated and stored in the cells without major changes in their structure, possibly through the rare property of direct encapsulation (Bati et al., 1984).

## ROLE OF METABOLIC ENGINEERING IN HYDROPHOBIC WASTES UTILIZATION BY OLEAGINOUS MICROORGANISMS: FUTURE PERSPECTIVES

**FIGURE 2:**
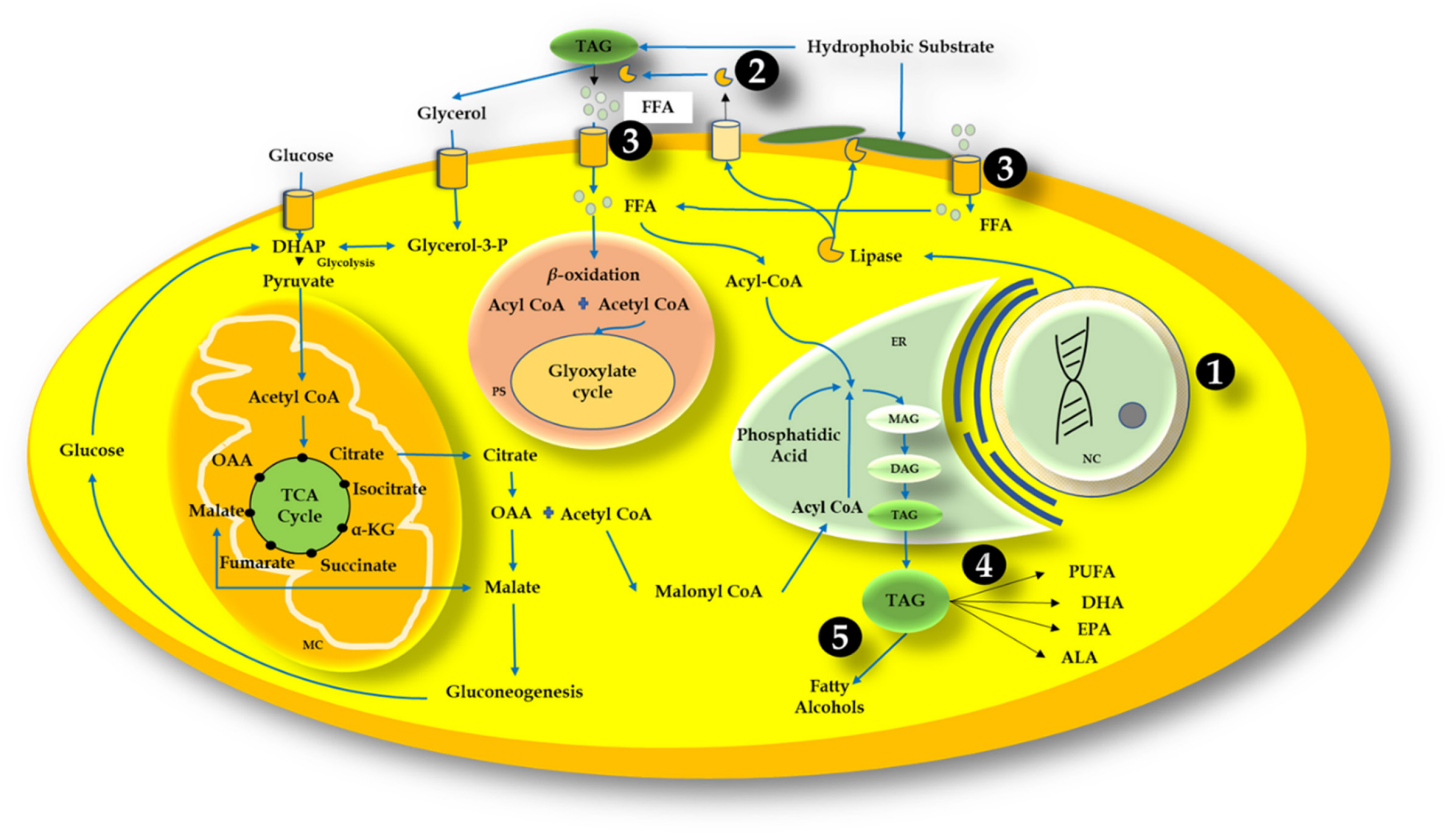
Role of metabolic engineering in the utilization of hydrophobic wastes by oleaginous microorganisms: Challenges and approaches (1) Missing gene annotation through advanced gene clustering; (2) Identification and characterization of potential novel lipase and lipolytic microorganisms; (3) Identification of mechanism and pathway for FFA transport; (4) Transformation into desired lipid profile using over‐expression or knock‐out of desaturase and similar enzymes; (5) Expression of enzymes related to fatty alcohol production. Adapted and modified from (Fabiszewska et al., 2022) [ALA, α‐linolenic acid; DAG, Diacylglycerols; DHA, Docosahexaenoic acid, EPA, Eicosapentaenoic acid; DHAP, Dihydroxy acetone phosphate; ER, Endoplasmic reticulum; FFA, Free fatty acids; MAG, Monoacylglycerols; MC, Mitochondria; NC, Nucleus; OAA, Oxaloacetic acid; PS, Peroxisome; PUFA, Polyunsaturated fatty acids; TAG, Triacyclglycerols; α‐KG, α‐ketoglutarate].

In recent years, omics and synthetic biology have attracted scientists' interest as potential areas for industrial applications. In eukaryotes, the metabolism of fatty substrates has evolved over a long period. From the extracellular degradation to the biotransformation of lipids, there are several important enzymes with mechanistic differences among the oleaginous microorganisms following different metabolic pathway routes and likely to be regulated differently at several branch points. As a result, such unidentified enzymes involved in the process are crucial targets for improving oleochemicals synthesis by using various metabolic engineering approaches. To begin, an omics‐based approach (transcriptomics, proteomics and metabolomics) should be used to understand the metabolic pathways and identify key enzymes and regulatory factors involved in the valorization of hydrophobic substrates. Using differential gene expression, omics analysis can provide critical information on physiological growth and other essential characteristics for future bioprocess studies. The second stage is to reconstruct the pathway using the rational alteration of fluxes to funnel down the desired products after their identification. The combination of insights into bioprocessing and molecular responses of cells, as well as the availability of various next‐generation genome modification techniques such as CRISPR/Cas9 and TALENs, has made it easier to develop novel single‐cell biorefineries with improved characteristics that can meet criteria for industrial use. EasycloneYALI is one such example which is a tool developed for streamlined strain improvement using CRISPR/Cas9 in the oleaginous yeast *Y. lipolytica* (Holkenbrink et al., 2018). Various bottleneck challenges and approaches using molecular biology are discussed in this section (Figure 2).

With the recent technological advancements, the genomes of the majority of microorganisms are sequenced and available on the public databases. A very first challenge is the limited or missing gene annotations of some important proteins in such microorganisms, and many of the proteins are currently annotated as hypothetical proteins, but instead, they might have a vital role in the metabolism process (Poorinmohammad & Kerkhoven, 2021). Recently novel genes encoding for extracellular lipases with unique class three features in the thraustochytrid species *A. limacinum* mh0186 were identified, cloned, and characterized using the comparative gene clustering method. This information was missing in the gene annotations due to no homology with existing lipases in the database (Ishibashi et al., 2019). The gene search for possible novel enzymes with experimental data support from the literature should be identified using advanced gene clustering and annotation tools followed by experimental validation of the function.

A second challenge is to identify oleaginous microorganisms exhibiting high lipolytic activities. Efficient high‐throughput screening techniques may be used to identify such potential candidates in the future. The genes responsible for 16 different lipases with variation in substrate specificity were identified along with characterization in the yeast *Y. lipolytica* (Fickers et al., 2005). More studies into understanding structural and functional insights about such enzymes using bioinformatics and laboratory‐scale studies will be helpful in the development of recombinant strains with improved lipolytic activity and fatty acid uptake. The novel lipase genes thus identified will also help in developing strains with over‐expressed enzymes for improved utilizations.

The third challenge is to understand the transport of free fatty acids from external environments to the intracellular organelles. Two possible fatty acid intracellular transport models were proposed in *Y. lipolytica* and suggested that fatty acid transport and activation proteins in this yeast such as aa1p, Pxa1p, Pxa2p and Ant1p were conserved from *Saccharomyces cerevisiae*. Despite the structural similarity among proteins, differences were observed in their functions in *Y. lipolytica* (Dulermo et al., 2015). It will also be interesting to study and identify the important proteins and enzymes that aid in the transport of extracellular fatty acids inside the cells (Dulermo et al., 2015). Understanding the intracellular transport of fatty acid in peroxisomes or mitochondria is also of great interest. With the help of omics, metabolic flux analysis and genetic engineering, more pathways will be identified in the future. A genome‐scale metabolic model will eventually help to unravel important pathways and future biotechnological interventions.

When fatty acids are available in the growth medium, the *de‐novo* pathway is blocked by the inhibition of important enzymes such as fatty acid synthase (FAS) and ATP‐Citrate lyase (ACL) (Papanikolaou & Aggelis, 2011; Tzirita et al., 2020). Oleochemicals such as DHA are synthesized via *de‐novo* synthesis and therefore it is challenging to select a microorganism that is capable of simultaneous *de‐novo* and *ex‐novo* synthesis. One such example is *Y. lipolytica* ACA‐DC 50109 which could also follow the *de‐novo* synthesis despite the presence of fatty acids (C16 and C18) in the external media (Papanikolaou et al., 2003). In a recent study, a thraustochytrid species *A. limacinum* SR21 could accumulate about 16% more DHA content when it was cultivated in the simultaneous *de‐novo* and *ex‐novo* mode using a combination of volatile fatty acids and waste cooking oil as substrate as compared to the medium containing a mixture of glucose and waste cooking oil (Patel, Delgado Vellosillo, et al., 2022b). Isolation and exploration for more novel species of thraustochytrids will aid in the valorization of wastes containing mixtures of hydrophilic and hydrophobic substrates for improved production.

Designing targeted tailoring of the fatty acid waste profile into appropriate value‐added lipids is a challenging process. Intracellular desaturases (as Δ_9_ and Δ_12_) are the enzymes that can potentially affect the overall saturation index of the lipid produced by introducing double bonds (Cook & McMaster, 2002). Over‐expression or knock‐out of such genes can provide the desired PUFA production during the biotransformation of external lipids. For instance, a 7.5‐fold increase in the α‐linoleic acid (ALA) was observed in the recombinant strain of *T. oleaginous* ATCC 20509. Also strains for higher PUFA accumulation as compared to the wild type were achieved through the expression of bi‐functional desaturase, elongase and ALA‐isomerase enzymes from different species into the yeast (Görner et al., 2016). Similarly, the expression of Δ‐9 fatty acid desaturase achieved higher oleic acid content in *Rhodosporidium toruloides* (Tsai et al., 2019). Such genetically modified microorganisms can save a lot of energy and downstream processing of oleic acid (or another component) enrichment and can be achieved directly in a single‐step fermentation process. Several desaturases and elongases have been identified in thraustochytrids species and recently reviewed in detail (Patel et al., 2021). Some of the efforts for over‐expression of Δ‐9 fatty acid desaturase in *A. limacinum* mh0186 increased EPA by 4.6‐fold, while also some strategies were applied to express exogenous desaturases for changes in DHA production by *Schizochytrium* sp. (Kobayashi et al., 2011; Ren et al., 2015). More such strategies can help tailor the biotransformation process to funnel down to important products relevant to industrial applications in the future.

Fatty alcohols are derived from the fatty acids generated and have importance in the industry as surfactants and emulsifiers. In a recent study, fatty acyl‐CoA reductase from *Mus musculus* was over‐expressed in *Lipomyces starkeyi* for the production of long‐chain fatty alcohol production (McNeil & Stuart, 2018). Similar studies have also been performed for other species such as *Y. lipolytica* (Cordova et al., 2020). More efforts are needed in the development of recombinant thraustochytrids species that can utilize potential sources of free fatty acids for the direct production of industrially important fatty alcohols in the future.

Finally, it is critical to follow all applicable laws and regulations for the use of genetically modified organisms, which vary by nation, as the products may be used for nutraceuticals or pharmaceuticals.

## CONCLUDING REMARKS

The amount of hydrophobic wastes produced will rise along with the population, and the current waste treatment techniques will not be adequate. Such wastes are potential substrates for their valorization into high‐value oleochemicals using oleaginous microbes. The industrial importance and market values of oleochemicals have drawn significant attention and are going to boost the industry in the next few decades due to their wide range of applications. It will be possible to explore thraustochytrids species at their full potential for large ranges of multi‐products through hydrophobic waste valorization considering recent advances in genetic engineering and bioprocessing in the future.

## CONFLICT OF INTEREST

The authors declare no conflict of interest.
